# Hyperbilirubinemia predicts the infectious complications after esophagectomy for esophageal cancer

**DOI:** 10.1016/j.amsu.2019.02.004

**Published:** 2019-02-21

**Authors:** Yusuke Muneoka, Hiroshi Ichikawa, Shin-ichi Kosugi, Takaaki Hanyu, Takashi Ishikawa, Yosuke Kano, Yoshifumi Shimada, Masayuki Nagahashi, Jun Sakata, Takashi Kobayashi, Hitoshi Kameyama, Kohei Akazawa, Toshifumi Wakai

**Affiliations:** a Division of Digestive and General Surgery, Niigata University Graduate School of Medical and Dental Sciences, 1-757 Asahimachi-dori, Chuo-ku, Niigata, 951-8510, Japan; b Department of Digestive and General Surgery, Uonuma Institute of Community Medicine, Niigata University Medical and Dental Hospital, 4132 Urasa, Minamiuonuma-shi, Niigata, 949-7320, Japan; c Department of Medical Informatics, Niigata University Medical and Dental Hospital, 1-754 Asahimachi-dori, Chuo-ku, Niigata, 951-8520, Japan

**Keywords:** Hyperbilirubinemia, Infection, Complications, Esophageal cancer, Esophagectomy, EC, Esophageal cancer, PICs, postoperative infectious complications, LN, lymph node, TNM, tumor-node-metastasis, UICC, International Union Against Cancer, OE, open transthoracic esophagectomy, MIE, Minimally invasive transthoracic esophagectomy, THE, Transhiatal esophagectomy, T-bil, total bilirubin, CRP, C-reactive protein, OR, odds ratio, CI, confidence of interval, WBC, white blood cell

## Abstract

**Background:**

Surgical stress and inflammation can cause hyperbilirubinemia, which sometimes occurs after esophagectomy for esophageal cancer (EC). The aim of this study was to elucidate the clinical significance of postoperative hyperbilirubinemia in the management of EC patients.

**Materials and methods:**

We retrospectively reviewed records of 81 EC patients who underwent esophagectomy from 2009 to 2014. We compared the clinicopathological and perioperative factors, including the presence of hyperbilirubinemia (total bilirubin ≥1.5 mg/dL), between patients with postoperative infectious complications (PIC group) and those without (Non-PIC group).

**Results:**

PIC developed in 52 patients (64.2%). There were significant differences in incidence of postoperative hyperbilirubinemia between the PIC group and the non-PIC group (34.6% vs. 3.4%, *P* = 0.002), as well as the approach of esophagectomy (*P* = 0.045), the surgical duration (469 vs. 389 min, *P* < 0.001), the amount of blood loss (420 vs. 300 mL, *P* = 0.018), the frequency of intraoperative blood transfusions (32.7% vs. 6.9%, *P* = 0.012) and the peak postoperative C-reactive protein level (17.3 vs. 8.6 mg/dL, *P* = 0.007). Multivariate analysis revealed hyperbilirubinemia was independently associated with the occurrence of PICs (odds ratio: 38.6, *P* = 0.010). The median time to the diagnosis of hyperbilirubinemia was significantly shorter than that of PICs (3.0 vs. 4.5 days, *P* = 0.025).

**Conclusions:**

Postoperative hyperbilirubinemia was associated with the occurrence of PICs and frequently occurred before any PICs become apparent. More attention should be paid to the serum bilirubin level in the management after esophagectomy for EC.

## Highlights

• Hyperbilirubinemia was related to the infectious complications after esophagectomy.
• Hyperbilirubinemia preceded the infectious complications became apparent.
• Total bilirubin level might be a useful indicator for the infectious complications.

## 1 Introduction

Esophageal cancer is the eighth most common cancer worldwide and was the sixth most common cause of cancer-related death in 2012 [1]. Esophagectomy with lymph node (LN) dissection is the principal treatment for locally advanced cancer. Although recent advances in surgical techniques and perioperative management have improved long-term outcomes after esophagectomy [2], esophagectomy remains an extremely invasive procedure with severe morbidity and high mortality. Nation-wide studies in Japan, France and United states showed 41.9–50.0% of morbidity and 1.2–5.6% of 30-day mortality after esophagectomy [[3], [4], [5]]. Research informing better management strategies for post-esophagectomy patients could reduce the morbidity and mortality associated with this procedure.

Postoperative infectious complications (PICs), such as pneumonia and deep organ space infection, represent a major cause of morbidity and mortality following esophagectomy, resulting in longer hospital stays and higher medical costs [3,6,7]. Recently, it has been suggested that the development of severe PICs is associated with adverse oncologic outcomes in esophageal cancer patients [8,9]. Given that severe PICs can be harmful to both short- and long-term outcomes after esophagectomy, early detection and appropriate management to prevent the aggravation of PICs are critical for achieving favorable outcomes in these patients.

Elevated serum bilirubin levels are sometimes observed in patients following esophagectomy for esophageal cancer. It has been shown that surgical stress and inflammation after major surgery cause postoperative hyperbilirubinemia [10]. In addition, several studies have elucidated that postoperative hyperbilirubinemia is associated with the extent of surgery and postoperative complications [11,12]. However, the clinical implication of postoperative hyperbilirubinemia in the management of these patients has not yet been clarified.

In this study we hypothesized that postoperative hyperbilirubinemia is an informative factor in the appropriate management of patients after esophagectomy for esophageal cancer. The aim of this study was to investigate the association between postoperative hyperbilirubinemia and the occurrence of PICs.

## 2 Materials and methods

### 2.1 Study population

We retrospectively investigated 81 consecutive patients with thoracic esophageal cancer who underwent curative esophagectomy with LN dissection between January 2009 and December 2014. This study has been reported in line with the Strengthening the Reporting of Cohort Studies in Surgery (STROCSS) criteria [13]. Clinical data from the medical records of these patients were retrospectively collected and reviewed. The primary tumor characteristics were described according to the tumor-node-metastasis (TNM) classification of the International Union Against Cancer (UICC), 7th edition [14]. The characteristics of the 81 patients are shown in Table 1. The study population comprised 63 (77.8%) men and 18 (22.2%) women, with the median age of 68 years (range, 44–82 years). Of the 81 patients, six patients had a history of chronic liver disease due to alcoholic chronic hepatitis.

**Table 1:** Patient and tumor characteristics.

Variables	N = 81
Age: median (range)	68 (44–82)
Gender	
Male/Female	63/18
BMI: median (range)	21.6 (14.5–25.7)
History of liver disease	
Absent/Present	75/6
Performance status (ECOG)	
0/1/2	53/26/2
Preoperative nutritional support	
Absent/Present	64/17
Preoperative chemotherapy	
Absent/Present	42/39
Preoperative T-bil level (mg/dL)	
Median (range)	0.5(0.1–1.8)
Preoperative serum Alb level (g/dL)	
Median (range)	3.9 (2.8–4.8)
Tumor location	
Ut/Mt/Lt	8/46/27
Histological type	
SCC/Ad/Other	75/2/4
UICC-pT stage	
T0/T1/T2/T3/T4	4^a^/37/11/25/4
UICC-pN stage	
N0/N1/N2/N3	42/23/12/4
UICC-pM stage	
M0/M1	79/2^b^
UICC-pStage	
0/I/II/III/IV	4/32/19/24/2

BMI, body mass index; ECOG, Eastern Cooperative Oncology Group.

T-bil, total bilirubin; Alb, albumin; Ut, upper thoracic; Mt, mid-thoracic; Lt, lower thoracic; SCC, squamous cell carcinoma; Ad, adenocarcinoma; UICC, International Union Against Cancer.

a These patients achieved complete response by preoperative chemotherapy.

b These patients had metastasis in supraclavicular lymph nodes.

### 2.2 Surgery and preoperative chemotherapy

Surgical procedures, including the approach and the extent of LN dissection, depended mainly on tumor location, preoperative staging, and patients’ general condition and comorbidities, as described elsewhere [15,16]. In brief, open transthoracic esophagectomy (OE) was a standard surgical procedure in our institution. Minimally invasive transthoracic esophagectomy (MIE) with thoracoscopic approach was performed for superficial carcinoma without mediastinal LN metastasis based on clinical staging. Transthoracic esophagectomy including OE and MIE were performed either with mediastinal and abdominal LN dissection (two-field LN dissection) or cervical, mediastinal, and abdominal LN dissection (three-field LN dissection). Transhiatal esophagectomy (THE) with abdominal and limited mediastinal LN dissection was performed for tumor of the lower thoracic esophagus without mediastinal LN metastasis. All of the patients underwent feeding jejunostomy at the same time of esophagectomy, and enteral nutrition was started within 4 days after esophagectomy. Preoperative chemotherapy using a combination of 5-fluorouracil and cisplatin or nedaplatin was performed in patients with stage IB, II or III disease.

### 2.3 Definition of postoperative hyperbilirubinemia and complications

We performed routine blood tests at postoperative days 1, 2, 3, 5 and 7. The subsequent blood tests were performed according to the clinicians’ decision. We defined postoperative hyperbilirubinemia as a peak in the total bilirubin (T-bil) level above the upper limit of normal (≥1.5 mg/dL) within 7 days following esophagectomy. If the occurrence of postoperative complications was suspected based on clinical symptoms and/or the results of the blood test, complete examinations, such as X-ray imaging and computed tomography, were added to diagnose complications. We defined the following complications which were grade II or higher according to the Clavien-Dindo classification [17] as PICs: pneumonia, superficial or deep incisional surgical site infection (SSI), organ/space SSI including anastomotic leakage, mediastinitis pyothorax, abdominal abscess, enteritis, blood stream infection, and urinary tract infection.

### 2.4 Data analysis

Differences of clinicopathological and perioperative factors between patients with postoperative infectious complications (PIC group) and those without (Non-PIC group) were assessed using the Mann-Whitney *U* test for continuous variables and the Pearson chi-square test or Fisher's exact test for categorical variables as univariate analyses. Differences in the number of days after esophagectomy until the diagnosis of postoperative hyperbilirubinemia and PICs were evaluated with the non-parametric Wilcoxon signed-rank test. A two-sided *P* value < 0.05 was considered statistically significant. Multivariate analyses were performed using logistic regression analyses to assess the independent association between postoperative hyperbilirubinemia and PICs: stepwise selection was used for variable selection, with entry and limits of *P* value < 0.05. The stability of this model was confirmed by means of a step-backward and step-forward fitting procedure. All analyses were performed using IBM SPSS statistical software version 22 (IBM Corp., Armonk, NY).

## 3 Results

### 3.1 The details of PICs and mortality in the study population

PICs occurred in 52 (64.2%) of 81 patients, and the details of PICs are shown in Table 2. The most common infectious complication was pneumonia, which developed in 31 patients (38.3%). Organ/space SSI was observed in 17 patients (21.0%), including anastomotic leakage in 12 patients (14.8%), mediastinitis in five patients (6.2%), pyothorax in one patient (1.2%), and abdominal abscess in one patient (1.2%). Mediastinitis and abdominal abscess caused by anastomotic leakage each occurred in one patient. Operative mortality was not observed in the study population.

**Table 2:** Postoperative infectious complications according to the Clavien-Dindo classification.

Variables	All patients (N = 81)				
	Grade I	Grade II	Grade III	Grade IV	Grade ≥ II
Any infectious complication	7	32	12	8	52 (64.2%)
Pneumonia	0	20	3	8	31 (38.3%)
Surgical site infection	7	13	11	0	24 (29.6%)
Superficial or deep incisional	7	5	2	0	7 (8.6%)
Organ/space	0	8^a^	9^b^	0	17 (21.0%)
Anastomotic leakage	0	6	6	0	12 (14.8%)
Mediastinitis	0	3	2	0	5 (6.2%)
Pyothorax	0	0	1	0	1 (1.2%)
Abdominal abscess	0	0	1	0	1 (1.2%)
Enteritis	1	7	0	0	7 (8.6%)
Blood stream infection	0	0	0	2	2 (2.4%)
Urinary tract infection	0	2	0	0	2 (2.4%)

Several infectious complications were observed in 20 patients.

a One patient had mediastinitis caused by anastomotic leakage.

b One patient had abdominal abscess caused by anastomotic leakage.

### 3.2 Clinicopathological and perioperative factors

There were no significant differences in any clinicopathological factors including the history of chronic liver disease, preoperative serum albumin and T-bil levels between the two groups (Table 3). The details of the perioperative data of the two groups are given in Table 4. The peak postoperative T-bil level was significantly higher in the PIC group than in non-PIC group (1.2 vs. 0.9 mg/dL, *P* < 0.001). The incidence of postoperative hyperbilirubinemia was significantly higher in the PIC group than in non-PIC group (34.6% vs. 3.4%, *P* = 0.002). In addition, there were significant differences between the PIC group and non-PIC group in the following five factors: the approach of esophagectomy (*P* = 0.013), the surgical duration (469 vs. 389 min, *P* < 0.001), the amount of blood loss (420 vs. 300 mL, *P* = 0.018), the frequency of intraoperative blood transfusions (32.7% vs. 6.9%, *P* = 0.012), and the peak postoperative C-reactive protein (CRP) level (17.3 vs. 8.6 mg/dL, *P* = 0.007). To assess the independent association between postoperative hyperbilirubinemia and the occurrence of PICs, the above-mentioned five factors and hyperbilirubinemia were included in the logistic regression analysis (Table 5). We found that hyperbilirubinemia (odds ratio [OR]: 38.6, 95% confidence of interval [CI]: 2.4–613.6) was independently associated with the occurrence of PICs, in addition to intraoperative blood transfusion (OR: 5.9, 95% CI: 1.1–33.2); elevated CRP level of 15 mg/dL or more, which was the median value of peak CRP levels within 7 days following esophagectomy in all cases (OR: 8.2, 95% CI: 2.0–33.8); and transthoracic esophagectomy (OR: 24.8, 95% CI: 3.4–180.3).

**Table 3:** Clinicopathological parameters in patients with postoperative infectious complications and patients without postoperative infectious complications.

Variables	PIC group	Non-PIC group	*P* value
	N = 52	N = 29	
Age: median (range)	70 (44–82)	68 (52–79)	0.972
Gender			0.173
Male/Female	43/9	20/9	
BMI: median (range)	21.5(14.5–25.6)	22.1(16.4–25.7)	0.798
History of liver disease			0.412
Absent/Present	47/5	28/1	
Performance status (ECOG)			0.279
0/1/2	37/14/1	16/12/1	
Preoperative nutritional support			0.961
Absent/Present	41/11	23/6	
Preoperative chemotherapy			0.631
Absent/Present	28/24	14/15	
Preoperative T-bil level (mg/dL)			0.054
Median (range)	0.6 (0.3–1.8)	0.5 (0.1–1.0)	
Preoperative serum Alb level (g/dL)			0.371
Median (range)	3.9 (2.8–4.6)	4.0 (3.3–4.8)	
Tumor location			0.266
Ut/Mt/Lt	6/32/14	2/14/13	
Histological type			0.819
SCC/Ad/Other	47/2/3	28/0/1	
UICC-pT stage			0.452
T0/T1/T2/T3/T4	2/21/8/19/2	2/16/3/6/2	
UICC-pN stage			0.146
N0/N1/N2/N3	25/14/11/2	17/9/1/2	
UICC-pM stage			0.535
M0/M1	50/2	29/0	
UICC-pStage			0.539
0/I/II/III/IV	2/18/12/18/2	2/14/7/6/0	

PIC, postoperative infectious complication; BMI, body mass index; ECOG, Eastern Cooperative Oncology Group; T-bil, total bilirubin; Alb, albumin; Ut, upper thoracic; Mt, mid-thoracic; Lt, lower thoracic; SCC, squamous cell carcinoma; Ad, adenocarcinoma; UICC, International Union Against Cancer.

**Table 4:** Perioperative parameters in patients with postoperative infectious complications and patients without postoperative infectious complications.

Variables	PIC group N = 52	Non-PIC group N = 29	*P* value
Approach of esophagectomy			0.013
Transthoracic^a^	46 (88.5%)	19 (65.5%)	
Transhiatal	6 (11.5%)	10 (34.5%)	
LN dissection			0.224
One/Two-field	30 (57.7%)	21 (72.4%)	
Three-field	22 (42.3%)	8 (27.6%)	
Number of dissected LN			0.059
Median (range)	57 (6–125)	38 (2–113)	
Surgical duration (min)			<0.001
Median (range)	469 (246–959)	389 (219–871)	
Blood loss (mL)			0.018
Median (range)	420 (95–1796)	300 (55–1150)	
Blood transfusion			0.012
Absent	35 (67.3%)	27 (93.1%)	
Present	17 (32.7%)	2 (6.9%)	
Peak WBC level ( × 10^4^/μL)			0.399
Median (range)	1.36 (0.56–3.30)	1.22 (0.80–2.32)	
Peak CRP level (mg/dL)			0.007
Median (range)	17.3 (1.08–34.9)	8.63 (0.75–33.1)	
Peak T-bil level (mg/dL)			<0.001
Median (range)	1.2 (0.5–4.3)	0.9 (0.5–2.2)	
Hyperbilirubinemia^b^			0.002
Abesent	34 (65.4%)	28 (96.6%)	
Present	18 (34.6%)	1 (3.4%)	

LN, lymph node; WBC, white blood cell; CRP, C-reactive protein; T-bil, total bilirubin.

a Transthoracic esophagectomy includes open esophagectomy (N = 37) and minimally invasive esophagectomy (N = 28).

b We defined hyperbilirubinemia as a peak in the total bilirubin level of ≥1.5 mg/dL.

**Table 5:** Multivariate analysis of risk factors for postoperative infectious complications.

Variables	Odds ratio	95% confidence interval	*P* value
Approach of esophagectomy			
Transhiatal	1.00		
Transthoracic^a^	24.81	3.42–180.30	0.002
Hyperbilirubinemia^b^			
Absent	1.00		
Present	38.56	2.42–613.59	0.010
Peak CRP level (mg/dl)			
< 15	1.00		
≥ 15	8.15	1.96–33.84	0.004
Blood transfusion			
Absent	1.00		
Present	5.94	1.06–33.17	0.042

CRP, C-reactive protein.

a Transthoracic esophagectomy includes open esophagectomy (N = 37) and minimally invasive esophagectomy (N = 28).

b We defined hyperbilirubinemia as a peak in the total bilirubin level of ≥1.5 mg/dL.

### 3.3 Time to diagnosis of hyperbilirubinemia and PICs after esophagectomy

**Fig. 1:**
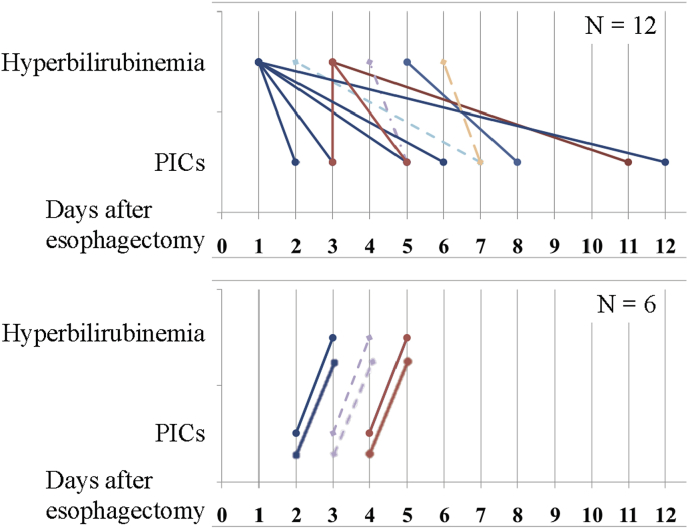
The number of days after esophagectomy until the diagnosis of hyperbilirubinemia and postoperative infectious complications. Upper graph shows 12 patients whose hyperbilirubinemia occurred before any PICs became apparent. Lower graph shows 6 patients whose hyperbilirubinemia occurred after any PICs became apparent. The median time to diagnosis of hyperbilirubinemia was significantly shorter than that of PICs (3.0 days vs. 4.5 days, *P* = 0.025).

To examine the clinical significance of postoperative hyperbilirubinemia, we focused on the 18 patients who developed PICs and hyperbilirubinemia after esophagectomy. The number of days after esophagectomy until the diagnosis of hyperbilirubinemia and PICs are shown in Fig. 1. Postoperative hyperbilirubinemia occurred before any PICs became apparent in 12 patients (67%) and the median time to the diagnosis of hyperbilirubinemia was significantly shorter than that of PICs (3.0 vs. 4.5 days, *P* = 0.025). On the other hand, the median time to the diagnosis of elevated CRP level over 15 mg/dL was almost same as that of PICs (4.0 vs. 4.5 days, *P* = 0.389).

## 4 Discussion

PICs such as pneumonia and anastomotic leakage unfortunately develop after esophagectomy in spite of meticulous postoperative management. Early detection and appropriate management to prevent the aggravation of PICs might be necessary to achieve favorable long-term outcomes after highly invasive esophagectomy with LN dissection for esophageal cancer. Several studies have already reported that hyperbilirubinemia is associated with not only major thoracic surgery such as esophagectomy but also post-esophagectomy complications [11,12]. We conducted this retrospective study to elucidate the clinical significance of postoperative hyperbilirubinemia especially in the management of patients with esophageal cancer after esophagectomy. Multivariate analysis revealed the significant association between hyperbilirubinemia within 7 days following esophagectomy and occurrence of PICs. Furthermore, postoperative hyperbilirubinemia occurred significantly earlier than any PICs became apparent in this patient population. To the best of our knowledge, this is the first study to demonstrate that T-bil level is a potentially useful marker for the early detection of PICs after esophagectomy for esophageal cancer.

The pathophysiologic mechanism of postoperative hyperbilirubinemia after esophagectomy has been conceived to be intrahepatic cholestasis due to cytokine overproduction. Geier et al. reported that proinflammatory cytokines, including tumor necrosis factor, interleukin-6, and interleukin-1β, which are produced by macrophages resident in the liver (Kupffer cells), were potent inhibitors of the hepatocellular and cholangiocellular transport systems to excrete bilirubin [18]. In the present study, the peak postoperative T-bil levels were significantly higher in the PIC group than in non-PIC group (1.2 vs. 0.9 mg/dL). PICs was the only independent risk factor of postoperative hyperbilirubinemia (data not shown, OR: 14.8, 95% CI: 1.86–118.05, *P* = 0.011). These results support the theory that cytokine overproduction and inflammation caused by PICs may be related to the elevation of postoperative bilirubin levels. Constitutional jaundice, as in Gilbert's syndrome and Dubin-Johnson syndrome, is also a known cause of postoperative hyperbilirubinemia [19]. In this study, the median preoperative T-bil level in the enrolled 81 patients was 0.5 mg/dL, and only one patient had preoperative T-bil level of 1.5 mg/dL or more. There were no significant differences in preoperative T-bil level between patients with PICs and those without it. Therefore, we considered that there was no potential association between constitutional jaundice and postoperative hyperbilirubinemia in this cohort.

In this study, we defined postoperative hyperbilirubinemia as a peak in T-bil level of 1.5 mg/dL or more within 7 days following esophagectomy. We could calculate T-bil cutoff level on the basis of sensitivity and specificity using receiver operator curve. However, there is a possibility that the calculated cutoff level excessively fit this research cohort and loses reproducibility. Therefore, we adopted the cutoff level of T-bil which was determined according to the upper limit value of the normal range (1.5 mg/dL) in our institution. In addition, because the routine blood tests were performed at postoperative days 1, 2, 3, 5 and 7, we could get the reliable data of T-bil without missing values within 7 days after esophagectomy in all the enrolled patients. Therefore, we used a peak T-bil level within 7 days after esophagectomy to define the postoperative hyperbilirubinemia.

At present several biochemical markers of the acute systemic inflammatory response, such as CRP and white blood cell (WBC) count, have been used for predicting postoperative complications in patients following surgery for esophagogastric cancer [20,21]. In line with these studies, elevated CRP level (≥15 mg/dL) was identified as one of the significantly associated factors with PICs in the present study. However, we found that the median time to the diagnosis of elevated CRP level was almost the same as that of PICs. On the other hand, the median time to diagnosis of postoperative hyperbilirubinemia as determined by T-bil testing was significantly shorter than that of PICs. In addition, the severity of PICs was not associated with whether the postoperative hyperbilirubinemia antecedent to PICs was observed or not (data not shown). These results suggest that T-bil might be a useful indicator for predicting PICs regardless of the severity in clinical practice.

In the 1990s the incidence of postoperative hyperbilirubinemia (T-bil level ≥2.0 mg/dL) was reported as 64–67% [[10], [11], [12],^22^]. A more recent study showed that the incidence of postoperative hyperbilirubinemia (≥2.0 mg/dL) was 35.5%, which was lower than the previous studies [23]. This discrepancy is likely due to the recent advances in operative techniques and perioperative management, which contribute to the reduction of postoperative inflammation. In this study the incidence of hyperbilirubinemia of 23.5% was lower still, even though the T-bil cutoff value of ≥1.5 mg/dL was lower than previous studies. One possible explanation of the lower incidence of hyperbilirubinemia might be the influence of early enteral nutrition. Early postoperative enteral nutrition from feeding jejunostomy is considered to reduce the production of inflammatory cytokines by preserving the gut mucosal barrier and preventing bacterial translocation [24,25]. Indeed, early enteral nutrition reduced the duration of systematic inflammatory response syndrome after esophagectomy in our previous study [26]. Aiko et al. showed that the mean values of total bilirubin were lower in the immediate enteral nutrition group than in the parenteral nutrition group [27]. Enteral nutrition was identified as an independent negatively associated factor with hyperbilirubinemia after esophagectomy [23]. The postoperative enteral nutrition was started within 4 days for all patients in our study. We carefully introduced the enteral nutrition with an initial dose of 200–250 mL of elemental diet under 20 mL/h to prevent the refeeding syndrome. On the other hand, patients who underwent total parental nutrition were included in the previous studies [10,12,23]. Therefore, postoperative early enteral nutrition might prevent the elevation of T-bil after esophagectomy.

There are three main limitations of the present study. First, this was a single institutional analysis of a small number of patients. However, we enrolled all the 81 consecutive patients who underwent curative esophagectomy for thoracic esophageal cancer during a short term from 2009 to 2014. This high rate and short-term inclusion period reduces potential selection bias arising from differences in preoperative management, surgical techniques, and preoperative therapies. Second, the traditional inflammatory factors such as IL-6 and IL-8 were associated with the complications after esophagectomy [[28], [29], [30]]. It is important to clarify a correlation between these factors and T-bil level. However, because we did not obtain the data on postoperative levels of these factors, we could not assess the correlation in this retrospective study. Future analysis should be conducted including this point. Third, it remains unclear whether early diagnostic approaches based on postoperative hyperbilirubinemia actually lead to the earlier detection and the reduction of the severe PICs and improvements in outcomes after esophagectomy. Nonetheless, we believe that our present findings are clinically informative for further multi-institutional prospective studies to demonstrate the clinical utility of T-bil level monitoring in the management following esophagectomy.

## 5 Conclusions

In conclusion, postoperative hyperbilirubinemia was independently associated with the occurrence of PICs in patients with esophageal cancer after esophagectomy. Furthermore, postoperative hyperbilirubinemia frequently occurred before any PICs become apparent. More attention should be paid to the serum bilirubin level in the management after esophagectomy for esophageal cancer.

## Ethical approval

The institutional review board at Niigata University Graduate School of Medical and Dental Sciences approved this study.

Judgment's number was 2016-0040.

## Sources of funding

This research was supported by the Japan Society for the Promotion of Science KAKENHI Grant Number JP16K10491.

## Author contribution

Study design: Yusuke Muneoka, Hiroshi Ichikawa and Shin-ichi Kosugi.

Data analysis: Yusuke Muneoka, Hiroshi Ichikawa.

Data collections: Yusuke Muneoka, Hiroshi Ichikawa, Takaaki Hanyu, Takashi Ishikawa and Yosuke Kano.

Supervised the study design and data analyses: Yoshifumi Shimada, Masayuki Nagahashi, Jun Sakata, Takashi Kobayashi and Hitoshi Kameyama.

Supervised the statistical analyses: Kohei Akazawa.

Supervised the whole study: Toshifumi Wakai.

Drafting the manuscript: Yusuke Muneoka, Hiroshi Ichikawa and Shin-ichi Kosugi.

Revising the manuscript, final approval of the version to be published: All authors.

## Conflicts of interest

The author have no financial conflicts of interest.

## Trial registry number

Our research is not RCT.

## Research registration unique identifying number (UIN)

UIN of this study was researchregistry4493.

## Guarantor

Hiroshi Ichikawa is the guarantor of this study.

## Provenance and peer review

Not commissioned, externally peer reviewed.
